# Executive Functions Are Associated with Fall Risk but not Balance in Chronic Cerebrovascular Disease

**DOI:** 10.3390/jcm9113405

**Published:** 2020-10-23

**Authors:** Cosimo Tuena, Valentina Mancuso, Ilaria M. A. Benzi, Pietro Cipresso, Alice Chirico, Karine Marie Goulene, Giuseppe Riva, Marco Stramba-Badiale, Elisa Pedroli

**Affiliations:** 1 Applied Technology for Neuro-Psychology Lab, IRCCS Istituto Auxologico Italiano, 20149 Milan, Italy; v.mancuso95@gmail.com (V.M.); imabenzi@gmail.com (I.M.A.B.); p.cipresso@auxologico.it (P.C.); giuseppe.riva@unicatt.it (G.R.); e.pedroli@auxologico.it (E.P.); 2 Department of Psychology, Università Cattolica del Sacro Cuore, 20123 Milan, Italy; 3 Università Cattolica del Sacro Cuore, 20123 Milan, Italy; alice.chirico@unicatt.it; 4 Department of Geriatrics and Cardiovascular Medicine, IRCCS Istituto Auxologico Italiano, 20149 Milan, Italy; goulene@auxologico.it (K.M.G.); stramba_badiale@auxologico.it (M.S.-B.); 5 Humane Technology Lab, Università Cattolica del Sacro Cuore, 20123 Milan, Italy; 6 Faculty of Psychology, Università eCampus, 22060 Novedrate, Italy

**Keywords:** vascular cognitive impairment, Trail Making Test, Bayesian statistics, Morse fall scale

## Abstract

Background: Older people’s deficits in executive functions (EF) have been shown to lead to higher fall risk, postural sway, and reduced speed. Crucially, EF impairments are even more pronounced in individuals with chronic cerebrovascular disease (CVD), namely vascular cognitive impairment. Methods: In this retrospective cross-sectional study, we used a complete neuropsychological battery, including the Trail Making Test (TMT) and physical measures, such as the Morse fall and EQUI scales, to assess 66 individuals with chronic CVD. Linear regressions, Bayesian analyses, and model selection were performed to see the impact of EF, global cognition, and vascular parkinsonism/hemiplegia on physical measures (fall risk and balance). Results: The TMT part B and BA correlated (*r* = 0.44 and *r* = 0.45) with Morse fall scale. Only EF significantly explained fall risk, whereas global cognition and vascular parkinsonism/hemiplegia did not. These findings were confirmed by Bayesian evidence and parsimony model selection. Balance was not significantly correlated with any of the neuropsychological tests. Conclusions: This is the first study investigating the relationship between cognitive and physical measures in a sample of older people with chronic CVD. The results are consistent with previous findings that link EF with fall risk in CVD.

## 1. Introduction

Cognition is an umbrella term that encompasses several domains (e.g., memory, attention, visuospatial ability, and executive functions [EF]) that contribute to processing information during functional tasks to maintain balance and prevent falls in older people [1]. EF is the cognitive domain responsible for monitoring, controlling, integrating, organizing, and maintaining internal and external information for achieving goals, decision-making, problem-solving, and modifying behaviors according to environmental demands [2,3,4]. Therefore, EF is vital to accomplishing complex tasks that require the coordination of various cognitive sub-components (e.g., attention, set-shifting, and working memory) [5]. Thus, these abilities are crucially related to balance and locomotion [2,4,6,7,8]. Balance and gait are not simple motor tasks but complex and goal-oriented activities that require constant awareness and interaction between body movements and the surrounding environment [5]. Even though several factors can explain the decline in balance and mobility in older people (e.g., vestibular, vision, biomechanical), research has also shown that age-related changes that occur in the prefrontal area can contribute to locomotion deficits [1,9]; thus, it has been suggested that impairments in the EF, located in that area, could be one of the significant triggers of those deficits [2,9]. Accordingly, executive dysfunctions and impairments in global functioning and memory are associated with higher fall risk and postural sway, and lower speed in this population [1,2,4,7]. When the demands of the simultaneous execution of a motor task (e.g., walking) increase cognitive load, the whole motor-cognitive performance could be affected [10]. Future fall risk is associated with gait changes under a dual-task compared to single-task testing [11]. Usually, a dual-task consists of walking activity and a non-walking task and requires specifically EF such as working memory and cognitive flexibility that are prone to deterioration with age [5]. Indeed, Hobert and colleagues [12] showed that healthy older people (50–78 years) with lower working memory and cognitive flexibility, assessed using the Trail Making Test (TMT) [13], presented higher dual-task costs in the walking task, compared to those with higher cognitive functions.

Likewise, older people with cognitive deficits are more vulnerable to physical mobility problems, with a higher fall risk than those without cognitive deficits [14]. A significant correlation was observed between EF and physical performance measures in a study investigating the relationship between EF and fall risk in community-dwelling older people; however, locomotion performances were explained by EF scores in regression analyses only in those with mild cognitive impairment [15].

Cerebrovascular disease (CVD) encompasses disorders that affect the brain’s blood supply and include chronic and acute vascular disease [16]. When chronic, CVD can present a broad spectrum of deficits, ranging from subjective cognitive decline to dementia [17,18]. Cerebrovascular impairment is more likely associated with both large (e.g., extensive vessel atherosclerosis) or small vessel disease (e.g., lacunar infarcts in the deeper white matter), but also with more focal cerebral infarctions (e.g., ischemic stroke) that affect the blood supply in both cortical and subcortical brain areas. Vascular cognitive impairment (VCI) [18] comprises different degrees of cognitive impairment related to chronic CVD, regardless of underlying pathology (e.g., multiple or single territorial or small infarcts, strategic infarcts, large or small vessel disease, or a combination of both).

Cognitive impairments are mostly caused by a disruption in frontal-subcortical pathways in the form of white matter lesions or microbleeds affecting long association fibers or pathways that connect cortical and subcortical regions; however, stroke and multiple cortical infarcts can also affect cognition [17]. Although the neuropsychological profile of VCI can be heterogeneous, research has revealed that people with CVD show deficits in processing speed, attention, and EF, whereas memory and visuospatial functioning are less affected [17,19,20,21,22]. Gait and balance problems are early features of vascular dementia and could also be determined by the presence of vascular parkinsonism (VP) [23] or hemiplegia in chronic post-stroke patients [24]. More specifically, many studies have shown a significant association between fall risk and lower TMT performance in cognitively normal and impaired individuals (e.g., [4,6,15]). The TMT is a neuropsychological assessment tool with high validity and reliability to detect EF deficits. It consists of two parts [13]: TMT-A measures visual search and motor speed, while TMT-B assesses attention, visual scanning, motor speed and coordination, mental flexibility, and working memory; TMT-BA (B minus A) is a central executive measure.

To our knowledge, little research has investigated the crucial intertwining of EF, measures of physical mobility (balance and fall risk), and cardiovascular disease (potential risk factors for CVD and VCI; [17]) [25,26] and no study has investigated this relationship in older people with CVD.

Thus, given that fall risk is a significant factor for VCI and executive dysfunctions, this study aims at retrospectively investigating more in-depth the specific associations between EF and physiotherapy measures of balance and fall risk in patients with CVD, also considering their global cognitive status. Moreover, it aims at providing useful insights for a tailored clinical intervention at our department in this population, evaluating the impact of EF on balance/fall risk scores while controlling for global cognitive deterioration and vascular parkinsonism.

This contribution stems from the following hypotheses: (1) among cognitive functions, EF have a unique association with measures of physical mobility; (2) among cognitive functions, EF is the unique significant predictor associated with physical mobility in individuals with CVD; motor deficits (e.g., VP or chronic plegia) could explain part of this link, whereas global cognition is uncertain. These findings will also be supported using Bayesian hypothesis testing (evidence in favor of H1 against H0 for each predictor) and model parsimony. To test our claims, we retrospectively analyzed clinical data of patients admitted to the hospital for cognitive and physical evaluation and rehabilitation.

## 2. Methods

### 2.1. Participants

A total of 66 patients (mean age = 78.89 years, *SD* = 6.88; mean education = 11.47 years, *SD* = 4.30; mean Mini-Mental State Evaluation (MMSE) = 25.07 points, *SD* = 3.43, range = 16.10–30; 19 females) referred to the Department of Geriatrics and Cardiovascular Medicine of the Istituto Auxologico Italiano from 2017 to 2019 were included in this retrospective study. Patients were not recruited for a particular research study but visited for clinical purposes: this resulted in the different use of tests/battery according to clinical aims. Data were collected in the neuropsychology clinic department’s database and selected based on diagnosis and acceptable missing rate for tests (<50% missing rate).

All the patients were required by the department physiatrists to have a neuropsychological examination at the neuropsychology clinic of the ward due to chronic CVD. CVD was diagnosed by medical reports of both licensed neuroradiologist (compute tomography (CT)/magnetic resonance imaging (MRI) scan) and licensed neurologist and/or physiatrist at Istituto Auxologico Italiano. Chronic CVD classification [18] was confirmed by the presence of any forms of lesion (atrophy, white matter lesions, small and large infarcts, hemorrhage, other), which are etiological features of potential post-stroke, subcortical ischemic vascular, multi-infarct (cortical), and mixed dementia, and generally of VCI [18]. Further, individuals with reported neurological comorbidity of VP (CVD with VP = 10), idiopathic normal-pressure hydrocephalus (INPH) (CVD with INPH = 1), hemiplegia (*n* = 13), neuropathies (*n* = 4), hypoesthesia (*n* = 3), and vision deficits due to nervous systems disorders (*n* = 3) at the neuropsychological evaluation were recorded. Exclusion criteria were diagnosis of idiopathic Parkinson’s disease, multiple sclerosis, confirmed primary diagnosis of neurodegenerative diseases other than CVD, traumatic brain injury with loss of consciousness, INPH without CVD, primary diagnosis of psychiatric illness, balance disorders due to non-neurological conditions, vestibular disorders, or recent history of orthopedic injury or surgery.

All participants gave informed consent to use clinical and demographics data before providing information. Table 1 shows the demographics and clinical data of the sample.

### 2.2. Neuropsychological Examination

As the complete dataset was composed of several neuropsychological and physical tests used for clinical purposes, we selected subjects whose missing rate was below 50%. After this selection, the neuropsychological battery considered for analysis was composed of measures of global cognition, such as the MMSE (cut-off < 22) [27] and the Clock Drawing Test (CDT) (cut-off age > 70 with high education < 3 or with low education < 6) [28]. EF were investigated through the Frontal Assessment Battery (FAB) (cut-off < 13.5) [29] and the TMT-BA for central executive (cut-off > 186) [30], attentive shift by means of TMT part B (cut-off > 282) [30], logical reasoning with Raven’s coloured progressive matrices (cut-off ≤ 17.5) [31], psychomotor speed with part A of the TMT (cut-off > 93) [30], phonetic (cut-off ≤ 16) and semantic (cut-off ≤ 24) proficiency with fluencies [32]. Visuospatial learning was assessed with the delayed Rey–Osterrieth Complex Figure (ROCF) (cut-off < 9.47) [33], short-term memory was assessed with the digit span forward (cut-off ≤ 4.26) [34], and, finally, constructional abilities were assessed with the copy ROCF (cut-off < 28.88) [33].

### 2.3. Physiotherapy Measures

Again, we included those tests with a missing rate below 50%. EQUI scale [35] is an eight-item scale validated for individuals with multiple sclerosis, which consists of a set of balance tasks (sit-up, stand with eye closed, stand with eyes closed with head extended, lean forward, pick-up, push, rotation, tandem stance) rated from zero (poor balance) to two (good balance). The Morse fall scale [36] is a six-item scale assessing the history of falling, secondary diagnosis, ambulatory aid, intravenous therapy/heparin lock, gait/transferring performance, and ambulation self-assessment. Items provide a unique score, where a score of 0–24 denotes no risk, a score of 25–50 indicates a low risk, and a score greater than 51 shows a high risk of falling.

### 2.4. Procedure

Patients admitted to the Department of Geriatrics and Cardiovascular Medicine of the Istituto Auxologico Italiano followed assessment and rehabilitative procedures in the day hospital regime. Evaluation using the tests mentioned above was planned according to the patient’s clinical profile. Tests were administered by a licensed neuropsychologist (EP) and the physiotherapist team of the department. The authors (CT and VM) retrospectively selected from the department dataset patients according to CVD diagnosis and available tests (missing rate < 50%).

### 2.5. Analyses

Relevant analyses were performed using R version 3.6.2 [37]. Specific packages used for the code were as follows: *mice* [38] for data imputation and non-imputed dataset, *sjstats* [39] for regression models, and *gvlma* [40] for linear regression assumptions check. TMT-B, TMT-BA, and Morse fall scale were square rooted to reduce positive skewness, whereas FAB and EQUI scale values were subtracted by the constant and square rooted to reduce negative skewness. Correlations were used to explore the linear associations and interpreted according to the conventional approach [41]; once assured of a linear distribution between the variables, we used linear regressions to see the impact of cognitive functions, global cognition, and VP (covariates) on physical measures. A significant minimum correlation coefficient of 0.25 between TMT and measures of physical mobility had been considered of interest [15]. In this study, we were only interested in studying cognitive–physical associations. Hence we expected low parameters (i.e., *r* and *R*^2^), as we did not include other vital factors crucial to studying posture and locomotion in aging [42]. A minimum required sample of 30 participants was established according to 10:1 predictor ratio for multiple regression [43].

First, we explored datasets with missing values; in this case, for both correlations and linear regressions, missing values were excluded as R’s default option. Then, to ensure results were not biased by missing information (missing at random, MAR), we imputed values with predictive mean matching with five datasets iterated five times with ridge equal to 0.001 to make the procedure more robust [38]. The Supplementary Figure (Figure S1) shows in red imputed values for each variable. Pearson correlations and linear regressions results were pooled among the five datasets created to extract parameters and *p*-values. Six patients attended the department twice within the three years (at least one year between evaluations) and two patients three times (one year among assessments). These data were considered independent for the analyses. In this study, the *p*-value significance threshold was set at α < 0.05.

Additionally, to confirm evidence of our results, we computed Bayesian information criterion (BIC) [44] of linear models of interest to compare null, main (neuropsychological function only), and full terms models (neuropsychological function plus MMSE and VP). Upper bound Bayes factor (BF) [45], an easy Bayesian alternative extracted from *p*-values, was instead calculated to provide evidence of the alternative over the null hypothesis concerning the predictors’ effect in the linear regressions. BF was also used to test the alternative vs. null hypothesis for correlations. Interpretation of BF followed Jeffreys’s interpretation rules [46]: no evidence, anecdotal, moderate, strong, and decisive evidence in favor of H1 for values greater than one or H0 for values lower than one.

## 3. Results

### 3.1. Unique Associations between Executive Functions and Physiotherapy Measure of Fall Risk

First, we looked at correlations between neuropsychological functions and balance (EQUI scale)/fall risk (Morse fall scale) measures. EQUI scale showed weak and non-significant correlations with the neuropsychological tests of interest. Thus balance measure was not considered in further analyses. Conversely, EF significantly correlated with fall risk and was further analyzed.

**Figure 1:**
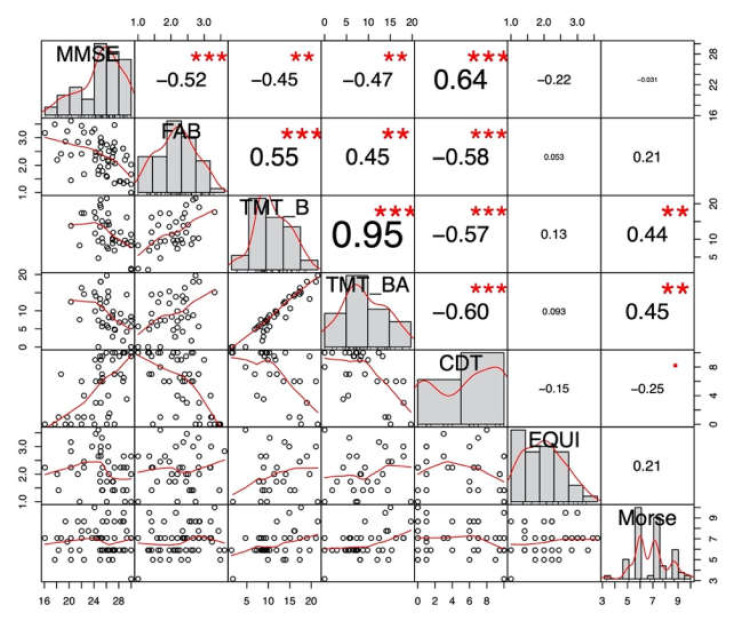
Non-imputed global cognition and executive functions correlations with physiotherapy measures. CDT: Clock Drawing Test; CTD: Cancellation Test of Digits; FAB: Frontal Assessment Battery; MMSE: Mini-Mental State Examination; TMT: Trail Making Test. *** *p* < 0.001; ** *p* < 0.001; * *p* < 0.05; *p* < 0.1.

Figure 1 shows significant correlations and their *r* values. Concerning our variables of interest, we found a significant moderate correlation between TMT-B and Morse scale (*r*(37) = 0.44, *p* < 0.01, 95% CI [0.14, 0.66]) and a significant moderate correlation between TMT-BA and Morse scale (*r*(37) = 0.45, *p* < 0.01, 95% CI [0.16, 0.67]). Then, five imputed datasets iterated five times were used for pooled correlations. Results showed weak positive correlations between TMT-B and Morse (*r* = 0.28, *p* < 0.05, 95% CI [0.01, 0.51]) and between TMT-BA and Morse (*r* = 0.31, *p* < 0.05, 95% CI [0.06, 0.53]). MMSE was not significantly correlated with Morse or EQUI scale and any other executive/attention or neuropsychological tests significantly correlated with Morse, hence other cognitive tests were not analysed in regression analyses.

### 3.2. The Impact of Executive Functions on Fall Risk Scores Controlling for Global Cognitive Deterioration and Vascular Parkinsonism

Assumptions of linear relations between variables, skewness, kurtosis, link function, and heteroscedasticity were satisfied for all the models tested. Two separate linear regressions were carried out to study the causal association of TMT-B and TMT-BA on fall risk, with MMSE and VP as covariates.

**Figure 2:**
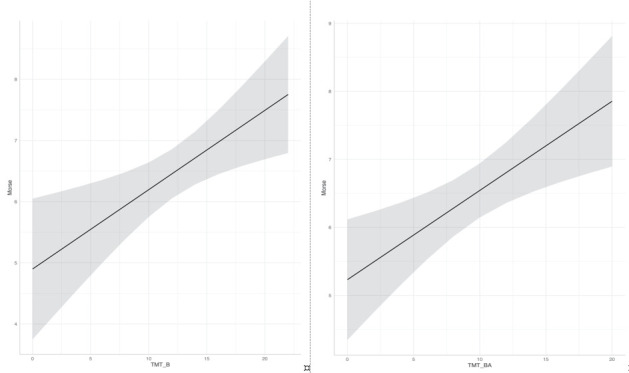
Causal association of TMT-B and TMT-BA square rooted scores on fall risk square rooted scores. TMT: Trail Making Test.

We found a significant linear regression (F(3,35) = 3.15, *p* < 0.05) with an *R*^2^ of 0.21. Participants’ fall risk score was equal to 4.26 + 0.13 (TMT-B) + 0.02 (MMSE) + 0.53 (VP). TMT-B was the only significant predictor (*p* < 0.01). Again, we found a significant linear regression (F(3,35) = 3.58, *p* < 0.05) with an *R*^2^ of 0.23. Participants’ fall risk score was equal to 4.24 + 0.13 (TMT-BA) + 0.04 (MMSE) + 0.61 (VP). TMT-BA was the only significant predictor (*p* < 0.01). We repeated the same analysis with the imputed dataset by pooling the results. Again, we found that TMT-B and TMT-BA were the only significant predictors (*p* < 0.05 and *p* < 0.01, respectively), although *R*^2^ decreased (0.10 and 0.13, respectively). Figure 2 shows the relationship of the fitted linear model for Morse scale and TMT-B and TMT-BA scores. Non-hemiplegic individuals were more frequent than hemiplegic (χ^2^ [1, *n* = 66] = 20.9, *p* < 0.001); nevertheless, we also checked whether hemiplegia at time of clinical evaluation could explain fall risk in place of VP. Again, TMT-B (*p* < 0.05) and TMT-BA (*p* < 0.01) were the only significant predictors.

### 3.3. Bayesian Correlations, Model Testing, and Effect Evidence

Significant correlations previously reported showed strong evidence (BF = 10 to 30) [46] in favor of the alternative compared to the null hypothesis; in particular, non-imputed TMT-B and Morse scores showed a BF of 10.7 and non-imputed TMT-BA and Morse scores a BF of 14.3. Table 2 shows beta values, standard error, *t*-values, and the upper bound BF for each parameter of the multiple regressions computed on the original dataset. Substantial evidence in favor of the predictors’ effect was found. The BIC for each null, main, and full model is reported; lower BIC values indicate the best model [44].

## 4. Discussion

Our study showed that (1) among the measures used, there is a unique association between EF and fall risk, confirming their role in locomotion in CVD as well and not only in healthy aging; any other neuropsychological functions correlate with balance or fall risk; (2) EF of shifting (TMT-B) and central executive (TMT-BA) are the only significant predictors that affect fall risk regardless of patients’ global cognitive status and parkinsonism or hemiplegia; this is also confirmed by strong Bayesian evidence and model parsimony testing. However, we must acknowledge that the strength of the linear regressions’ associations and determination coefficients showed low values. This could be since locomotion is a complex task that involves a broad range of factors (cognitive, clinical, sensorimotor, and physical functions) affected by aging [42]. In this study, we were uniquely interested in exploring cognitive–physical associations in CVD, and we did add other relevant measures associated with locomotion and posture in our analyses.

Our results align with previous findings that link EF with fall risk in older people and dementia patients, consolidating EF’s role during a motor activity such as locomotion [1,2,4,5]. Mainly, EF subcomponents of TMT have been consistently found to be involved in cognitive-motor processes related to fall risk (e.g., gait speed, mobility) and particularly in dual-tasking and motor control [2,4,5,11,15,25]. For instance, in Van Iersel and colleagues’ study [4] on healthy older people, TMT ratio (part B- part A/part A), but not Stroop test, affected gait performance, whereas TMT-BA and TMT B were found to be associated with speed and timed up and go tests [2,15]. In Blackwood’s study [25], the TMT-B’s relationships with gait speed and timed up and go test were also found in individuals with cardiovascular disease and normal cognitive status.

Interestingly, we did not find any associations between EF and the EQUI scale [35]. This might be related to the fact that this scale was validated on individuals with multiple sclerosis. Hence, it may not be valid for assessing balance in CVD people with subtle and different deficits. However, previous research showed that balance is not associated with EF but rather with global cognitive functioning [47].

Although memory and visuospatial functions in healthy aging could also be related to fall risk [1,48], our study did not find any other associations, probably because we considered people with CVD. Notably, EF and attention are significantly affected in VCI, whereas memory and visuospatial functions are typically less impaired [17,22]. Interestingly, we found that CVD’s fall risk is uniquely explained by EF but not by global cognition nor VP/hemiplegia. On the one hand, global cognition has been reported to affect fall risk in mild cognitive impairment but not in healthy older people [15], but this finding is not consistent in the literature [1]; on the other hand, it is surprising that the presence of hemiplegia did not affect fall risk, as this condition leads to locomotion deficits in individuals with stroke [24]: we explain this due to the imbalance of groups for this predictor. Interestingly, VP did not affect Morse fall scale, as VP is a predictor of falls and fractures [49]. However, given that people with VP are characterized by a frontal/dysexecutive profile [23,49], it might be the EF deficit and not the diagnosis per se related to fall risk. However, also, in this case, groups were severely imbalanced.

Finally, the standardized trend of EF and global cognition showed that it is crucial to design tailored EF rehabilitation in people with CVD regardless of their cognitive status. This is consistent with EF’s pivotal role in predicting rehabilitation outcomes in patients with CVD [50]. In particular, virtual reality could be used as a safe, standardized, tailored, and ecological tool to tap cognitive-physical dual-task and EF [51,52].

This contribution can be better understood in the context of its several limitations. Concerning our results, we must acknowledge that our analyses’ coefficients are weak, and the findings must be taken as preliminary. The relationship between EF and fall risk could be improved when adjusting for other factors (not controlled here) related to locomotion. Moreover, categorical predictors of VP and hemiplegia are not balanced, and results concerning their role in fall risk must be taken with caution. Regarding data collection, we did not include a control group or detailed CVD localization (e.g., subcortical, cortical, right, left). We did not have measures of autonomy (e.g., ADL/IADL) to differentiate mild VCI from dementia. However, previous research has consistently shown the link between EF and locomotion in older people [1]. We wanted to directly look at this pattern in VCI regardless of its severity or subtype since EF and locomotion issues are reported in these individuals [17,23]. Due to missing data, we could not include other neuropsychological measures of auditory-verbal learning or working memory (e.g., digit backward). However, a previous study [48] showed that delayed ROFC was loaded on the same “memory” principal component with auditory-verbal learning; lastly, working memory in our study was evaluated using the TMT-BA (central executive) [13], and we did not expect visuospatial or phonological working memory involvement [1]. Future studies could deepen the study of EF and locomotion in CVD by controlling other relevant locomotion and posture elements. Also, further research may want to investigate VCI cardiovascular risk factors [17] and how they relate to fall risk, even considering the effect of medication use and chronic motor/sensory deficits; as we did not have longitudinal data, we preferred to exclude this information to reduce confounding variables. More, it could be interesting to study also psychological factors, such as personality or attachment, in aging and CVD and how they relate to neurocognitive and sensorimotor performance [53,54,55,56,57]. Finally, concerning the rehabilitation of EF and locomotion, future studies could deepen the interaction of these two functions and investigate their impact on CVD’s rehabilitation path.

All in all, our research could be considered a preliminary study on patients with CVD, and additional clinical variables could be included in future works. Future studies should also compare the VCI and control groups and explore EF’s neural underpinnings and fall risk in this sample. Advanced predictive statistical techniques, such as machine learning, could be applied to improve diagnostic, prognostic, and therapeutic analyses.

## 5. Conclusions

In conclusion, no previous study explored EF and fall risk in people with CVD to the best of our knowledge. Despite providing preliminary data, our research shows a possible effect of EF on fall risk that should be studied in future investigations. This is also confirmed by advanced statistical techniques that support our analyses. Rehabilitation of EF in individuals with CVD with different degrees of VCI should be embedded in standard rehabilitative procedures to improve both cognitive (EF particularly) and motor performances.

## Figures and Tables

**Table 1:** Demographics and clinical characteristics of the groups.

	CVD (*n* = 66)	Missing Data
**Demographics**		
Age	78.89 (6.88)	0%
Education	11.47 (4.30)	0%
Gender	F = 19; M = 47	0%
**Neurological Comorbidities**		
VP	10 (15.2%)	0%
INPH	1 (1.5%)	0%
Hemiplegia	13 (19.7%)	3%
Hypoesthesia	3 (4.5%)	3%
Neuropathies	4 (6.1%)	3%
Vision	3 (4.5%)	3%
**Medical History**		
Hypertension	28 (42.2%)	4.5%
Diabetes	18 (27.3%)	3%
AF	15 (22.7%)	3%
CHD	13 (19.7%)	3%
History of Previous CVD	53 (80.3%)	3%
Hypercholesterolemia	0 (0%)	3%
**Main Current Medications**		
Patients with Medication	8 (12.1%)	3%
● SSRI	8 (100%)	0%
● ACE Inhibitor	4 (50%)	0%
● Beta-Blockers	3 (37.5%)	0%
***Cognitive Tests***		
● Global Cognition		
MMSE	25.07 (3.43)	0%
CDT	5.56 (3.78)	21.2%
● Executive Functions/Attention		
FAB	13.18 (3.11)	10.6%
TMT-B	163.55 (119.79)	39.3%
TMT-BA	109.65 (101.67)	39.3%
● Reasoning		
Raven	26.41 (5.87)	28.7%
● Psychomotor Speed		
TMT-A	64.59 (45.86)	25.7%
● Language		
honetic Fluency	24.05 (12.06)	6%
Semantic Fluency	28.74 (10.84)	7.5%
● Memory		
Delayed ROCF	13.88 (4.84)	24.2%
Digit Span Forward	5.17 (1.15)	45.4%
● Visuospatial		
Copy ROCF	26.51 (9.25)	24.2%
**Physiotherapy Tests**		
EQUI Scale	12.04 (3.10)	31.8%
Morse Fall Scale	48 (18.45)	1.5%

Data are reported with mean (standard deviation) or frequencies (%); ACE: angiotensin-converting-enzyme; AF: atrial fibrillation; CDT: Clock Drawing Test; CHD: coronary heart disease; F: females; FAB: Frontal Assessment Battery; INPH: idiopathic normal-pressure hydrocephalus; M: males; MMSE: Mini-Mental State Examination; ROCF: Rey–Osterrieth Complex Figure; TMT: Trail Making Test; SSRI: selective serotonin reuptake inhibitors; VP: vascular parkinsonism.

**Table 2:** Results of the linear regressions on fall risk and Bayesian analyses.

	β	Std. Error	*t*	*p*-Value	Upper Bound BF	BIC
Attentive shift model						
intercept	4.27	2.64	1.61	0.115		
TMT-B	0.13	0.04	2.87	0.007	10.77 **	
MMSE	0.02	0.09	0.27	0.790	1.99	
VP	0.54	0.59	0.91	0.368	1	
Null						227.40
Main						**132.16**
Full						138.45
Central executive model						
intercept	4.24	2.56	1.66	0.106		
TMT-BA	0.13	0.04	3.08	0.004	16.78 **	
MMSE	0.04	0.09	0.42	0.677	1.4	
VP	0.61	0.58	1.04	0.304	1.02	
Null						227.40
Main						**131.45**
Full						137.34

BF: Bayes factor; BIC: Bayesian information criterion; MMSE: Mini-Mental State Examination; TMT: Trail Making Test; VP: vascular parkinsonism. BF interpretation: ** strong evidence in favor of H1; Bold BIC values show the most parsimonious model.

## Supplementary Materials

The following are available online at https://www.mdpi.com/2077-0383/9/11/3405/s1: Figure S1: Imputed values with mice.
