# Supplementary figures and images for: Executive Functions Are Associated with Fall Risk but not Balance in Chronic Cerebrovascular Disease
Source: J Clin Med. 2020 Oct 23;9(11):3405. doi: 10.3390/jcm9113405 (PMC7690867; doi:10.3390/jcm9113405)

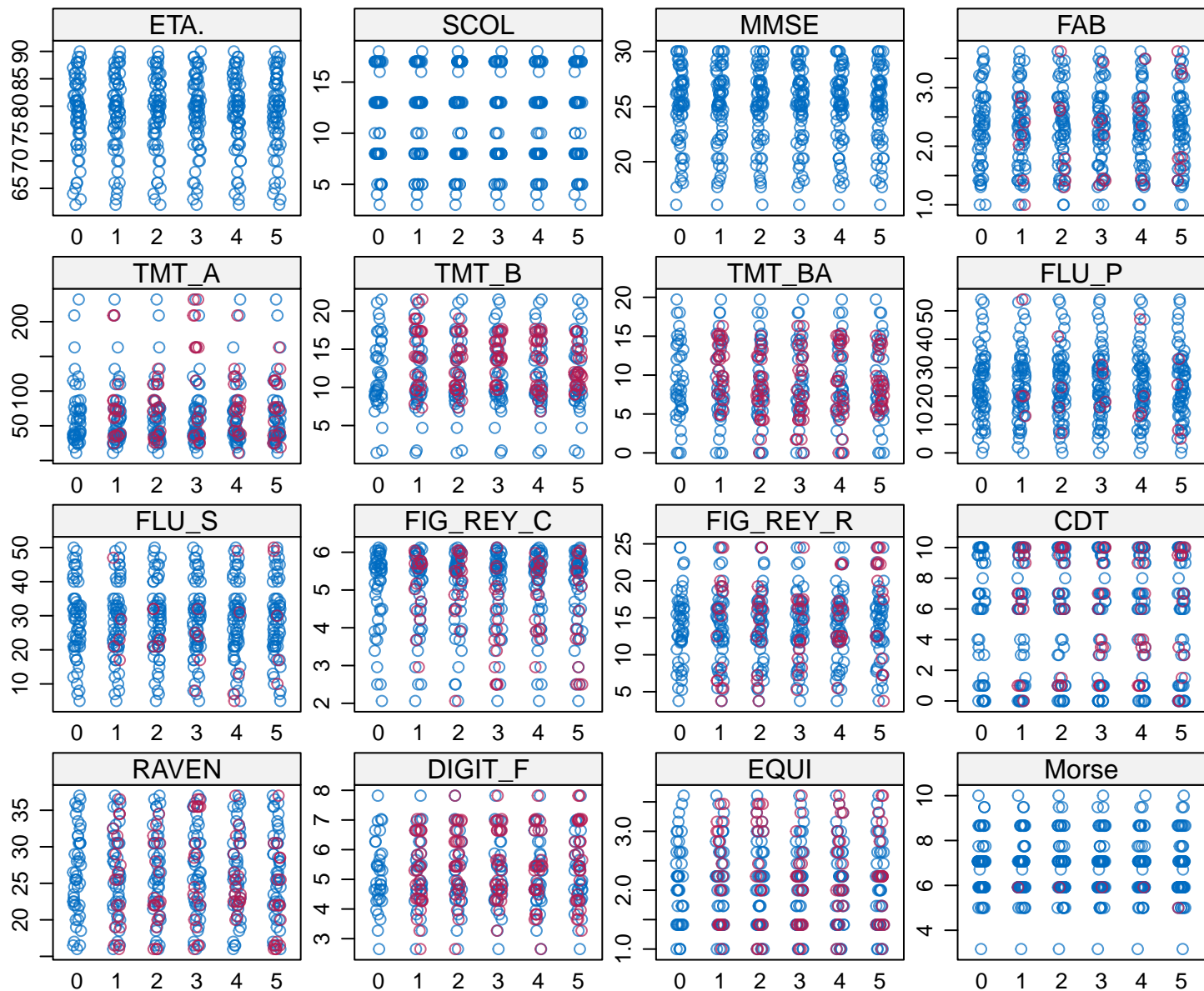

Supplement: Supplementary file 1 [file jcm-09-03405-s001.pdf]
